# Proteomic mapping of *Drosophila* transgenic elav.L-GAL4/+ brain as a tool to illuminate neuropathology mechanisms

**DOI:** 10.1038/s41598-020-62510-0

**Published:** 2020-03-25

**Authors:** Athanassios D. Velentzas, Stamatia A. Katarachia, Niki E. Sagioglou, Maria M. Tsioka, Athanasios K. Anagnostopoulos, Vassiliki E. Mpakou, Eleni I. Theotoki, Aikaterini F. Giannopoulou, Konstantinos E. Keramaris, Issidora S. Papassideri, George Th. Tsangaris, Dimitrios J. Stravopodis

**Affiliations:** 1 Section of Cell Biology and Biophysics, Department of Biology, School of Science, National and Kapodistrian University of Athens (NKUA), Athens, Greece; 2 Systems Biology Center, Biomedical Research Foundation of the Academy of Athens (BRFAA), Athens, Greece; 3 Department of Hematology and Bone Marrow Transplantation, Medical School, National and Kapodistrian University of Athens (NKUA), Athens, Greece

**Keywords:** Ageing, Mechanisms of disease

## Abstract

*Drosophila* brain has emerged as a powerful model system for the investigation of genes being related to neurological pathologies. To map the proteomic landscape of fly brain, in a high-resolution scale, we herein employed a nano liquid chromatography-tandem mass spectrometry technology, and high-content catalogues of 7,663 unique peptides and 2,335 single proteins were generated. Protein-data processing, through UniProt, DAVID, KEGG and PANTHER bioinformatics subroutines, led to fly brain-protein classification, according to sub-cellular topology, molecular function, implication in signaling and contribution to neuronal diseases. Given the importance of Ubiquitin Proteasome System (UPS) in neuropathologies and by using the almost completely reassembled UPS, we genetically targeted genes encoding components of the ubiquitination-dependent protein-degradation machinery. This analysis showed that driving RNAi toward proteasome components and regulators, using the GAL4-elav.L driver, resulted in changes to longevity and climbing-activity patterns during aging. Our proteomic map is expected to advance the existing knowledge regarding brain biology in animal species of major translational-research value and economical interest.

## Introduction

*Drosophila* nervous system has emerged as an effective and powerful biological model for the investigation of genes involved in human neurological pathologies, including Alzheimer’s, Amyotrophic Lateral Sclerosis (ALS), Huntington’s and Parkinson’s diseases^1^. Most of them are characterized by age-dependent deterioration in movement coordination and anatomical disruption in specific brain regions^2–5^. *Drosophila* and human are known to present a high genetic conservation pattern, with approximately 75% of all identified human disorder-related genes having fly homologues^1,6–9^. Compared to the roughly 86 billion neurons of the human brain^10^, the entire *Drosophila* adult central nervous system of 150,000 neurons^11,12^ provides a manageable number of cells, with a great diversity of distinguishable neuronal types, and, thus, an alternative research system for identification of the genetic and neurobiological basis of a wide array of human diseases^13,14^. In the same context, flies typically contain a single gene ortholog, in contrast to the multiple gene paralogs found in mammals. Most importantly, *Drosophila* offers a powerful genetic toolbox containing transgenic methods that enable the genetic perturbation of defined neuron sets^15^. Another advantage is the collection of approximately 7,000 transgenic fly lines which encompass (in a variety of intersecting patterns) all neurons of the brain^11,16^. Furthermore, a complete, at synaptic-resolution, electron microscopy dataset of adult fly whole-brain^17^, behavioral models for habituation^18^, and a growing number of antibodies all critically contribute to the knowledge advancement of brain biology and science.

Several studies have previously shown that the physiological operation of Ubiquitin Proteasome System (UPS), one of the two major protein degradation machineries, is essential for the proper formation of neuronal networks, and for the synaptic development and plasticity^4,19–21^. Aberrations in UPS are associated, either as primary causes or secondary consequences, with development of a number of neurological pathologies, such as ALS, Alzheimer’s, Huntington’s and Parkinson’s diseases, mainly due to the accumulation of aggregation-prone neurotoxic proteins^1,3,4,22–24^.

UPS is indispensable for the maintenance of protein homeostasis, since more than 80% of all cellular proteins are degraded through its proteolytic actions. Thus, proteasomes are the second most abundant protein complexes in cells, constituting up to 5% of the total protein content^25–28^. Elimination of ubiquitinated short-lived, misfolded and damaged proteins is mainly performed by the 26S proteasome. For their recognition and proteolysis by proteasome (26S), target proteins are reversibly tagged with a poly-ubiquitin chain via the sequential actions of E1 (ubiquitin-activating), E2 (ubiquitin-conjugating) and E3 (ubiquitin-ligase) enzymes^29,30^. The reverse process of ubiquitin (Ub) removal from Ub-bound proteins is accomplished by a superfamily of more than 100 deubiquitinating enzymes (DUBs)^27,31^. 26S proteasome is composed of a 20S proteolytic complex (core particle/CP), being capped by one or two 19S regulatory complexes (regulatory particle/RP)^28,32^. 20S-CP comprises 14 different α- and β-type subunits, stacked in 4 heptameric rings being arranged in an α1–7, β1–7, β1–7 and α1–7 architecture. The β1, β2 and β5 subunits possess caspase-, trypsin- and chymotrypsin-like activities, hydrolyzing proteins after acidic, basic and hydrophobic amino acid residues, respectively. Two outer α-rings confer attachment sites for the 19S-RP and form a molecular gate through which proteins enter the catalytic site^33,34^. 19S-RP carries 6 Rpt (Regulatory particle of triple-ATPase) and 13 Rpn (Regulatory particle of non-ATPase) subunits, and is responsible for recognition, binding, deubiquitination, unfolding and translocation of poly-ubiquitinated proteins to 20S-CP, in an ATP-dependent manner^26,27,33^. It (19S-RP) can be further divided into base and lid sub-complexes. Base, which is associated with 20S proteasome, is structured by the ATPases Rpt1–6, the scaffolding proteins Rpn1 and 2, and the ubiquitin receptors Rpn10 and 13. Lid, which recognizes, binds and deubiquitinates substrate proteins, is assembled by the Rpn3, 5–9, 11 (a metalloprotease with DUB activity), 12 and 15 subunits^27,33,35^.

Ιn the present study, through employment of a nano Liquid Chromatography - tandem Mass Spectrometry (nLC-MS/MS) proteomics approach, we have identified a total of 2,335 individual proteins in the single-transgenic elav.L-GAL4/+ female adult (2–3-day-old) *Drosophila* brain. This high-content and -reliability proteome database, and its subsequent bioinformatics processing, will certainly advance the existing knowledge on brain physiology and neurodegeneration pathology, and will also provide a powerful and versatile tool for future proteome comparisons among species of strong translational-research and economical interest. Moreover, and since the contribution of specific UPS components and regulators to normal brain function remains largely unknown, we have also examined the *in vivo* effects of gene-specific UPS disruptions in *Drosophila* neuronal tissues, after engagement of the GAL4/UAS binary genetic system suitably coupled to the RNAi-based technology, and use of lifespan and climbing activity profiles as the biological system readouts. Our study revealed that genetic targeting of UPS leads to the development of component/regulator-specific neuropathology.

## Materials and Methods

### Drosophila melanogaster strain stocks and culturing conditions

The *Drosophila melanogaster* transgenic fly strains w[*]; P{w[+mC] = GAL4-elav.L}3 (BL: 8760) and w[1118]; y[1] w[*]; w[*]; P{w[+mC] = UAS-UBP2.D}2/CyO (BL: 9907) were obtained from Bloomington *Drosophila* Stock Center (NIH P40OD018537) (IN, USA). The *D. melanogaster* transgenic fly strains UAS-Rpn2_RNAi (VDRC ID: 44135), UAS-alpha5_RNAi (VDRC ID: 16105), UAS-dbeta5_RNAi (VDRC ID: 38659), UAS-beta6_RNAi (VDRC ID: 34801) and UAS-UbcD1_RNAi (VDRC ID: 26011) were provided by Vienna *Drosophila* Resource Center (Vienna, Austria)^36^. Fly stocks were maintained at 25 °C, on a 12 h light/dark cycle, and fed on standard diet (6.4% rice flour, 5% tomato paste, 3.2% sugar, 0.8% yeast, 0.8% agar, 0.4% ethanol and 0.4% propionic acid).

### High-resolution proteοmics: peptide generation - nLC-MS/MS

Total-protein extracts were prepared from 100 *D. melanogaster* whole-brains, having been isolated, via manual dissection, from single-transgenic elav.L-GAL4/+ young adult (2–3-day-old) female flies. Protein extraction and processing were carried out as previously described^37^. Generated peptides were analyzed using an LTQ Orbitrap Elite instrument (Thermo Scientific, IL, USA), with the mass spectrometer being coupled to a Dionex Ultimate 3000 HPLC system. The extracted ion chromatogram was further processed using the Proteome Discoverer software (Thermo Scientific, IL, USA) and the Sequest search engine. The database chosen for protein identification searches was the *D. melanogaster* reference proteome, directly downloaded from UniProt 2.16 resource, without further modifications. Identification criteria included a precursor-mass tolerance of 10 ppm and fragment-mass tolerance of 0.05 Da. Trypsin was selected as the cleavage enzyme, with a maximum of “0” missed-cleavage parameter. A false-discovery rate-threshold of 0.5% ensured the reliability of protein identification procedure.

### Longevity measurement

Populations of 20–25 flies (males and females in separate vials) were thoroughly analyzed in terms of their respective longevities. Survival curves were generated by daily counting the number of deceased flies. The results of each viability experiment consisted of at least 100 flies, from three different fly crosses, were statistically analyzed. All viability experiments were performed at the same time for control and RNAi-downregulated strains.

Intriguingly, ubiquitous activation of RNAi machinery, through GeneSwitch system, has been previously reported to result in RNAi sequence-independent side-effects on *Drosophila* lifespan under aging^38^. However, RNAi expression restricted to certain tissues may not be detrimental to lifespan^38^. In accordance, we have recently shown, through engagement of the elav.L-GAL4/UAS and RNAi genetic platforms, that neuronal cell-specific targeting of *CCS* (copper chaperone for SOD1) gene affected climbing activity of *Drosophila* female flies during aging but not their longevity^39^, therefore suggesting that -at least- in this system the activation of RNAi machinery alone cannot affect aging.

### Climbing activity

Climbing activity (negative geotaxis assay) is a powerful *in vivo* indicator for the reliable evaluation of locomotor performance and, thus, neuromuscular integrity in *Drosophila*^40,41^. Climbing assay was carried out as previously described^42^. Briefly, 25–30 flies (males and females were being tested separately), from each experimental group, were being placed every 10 days in an empty graduated 100 ml (clean) cylinder, with a line drawn at the 66 ml (2/3) mark. Next, flies were gently tapped to the bottom of the cylinder, in order to start climbing (against gravity) all together. The number of flies that reached above the 66 ml mark, after a 20 sec time-period, was recorded. The assay was repeated 10 times for each group, allowing for 1 min rest-period in between 2 successive trials. Results were, then, converted to percentages, and the average pass rate per genotype and time point was computed. For each examined genotype, at least 5 different groups, from independent genetic crosses, per time-point were analyzed. Comparisons between control and RNAi-targeted fly-groups were carried out at the same time.

### Statistical analysis

Statistical analysis was performed using the Statistical Package for Social Sciences (IBM SPSS v23.0 for Windows IBM Corp., NY, USA). For climbing assays, the results were presented graphically as an average pass rate per genotype/time-point with sample standard deviation (±SSD) value. Differences between compared genotypes were evaluated using the independent *t*-test analysis. All data from lifespan experiments were analyzed with the Kaplan-Meier survival test, using log rank and Breslow test statistics. Significance was accepted at *p* < 0.05 (*) and *p* < 0.01 (**).

### Bioinformatics subroutines

The obtained UniProt^43^ protein accession numbers were processed for annotation through the DAVID bioinformatics resource (versions 6.7 and 6.8)^44,45^, the KEGG pathway maps^46–48^ and the PANTHER bioinformatics platform^49,50^.

## Results and Discussion

### High-resolution mapping of *Drosophila melanogaster* brain proteome: organelle compartmentalization and functional dissection of the mapped proteins

**Figure 1:**
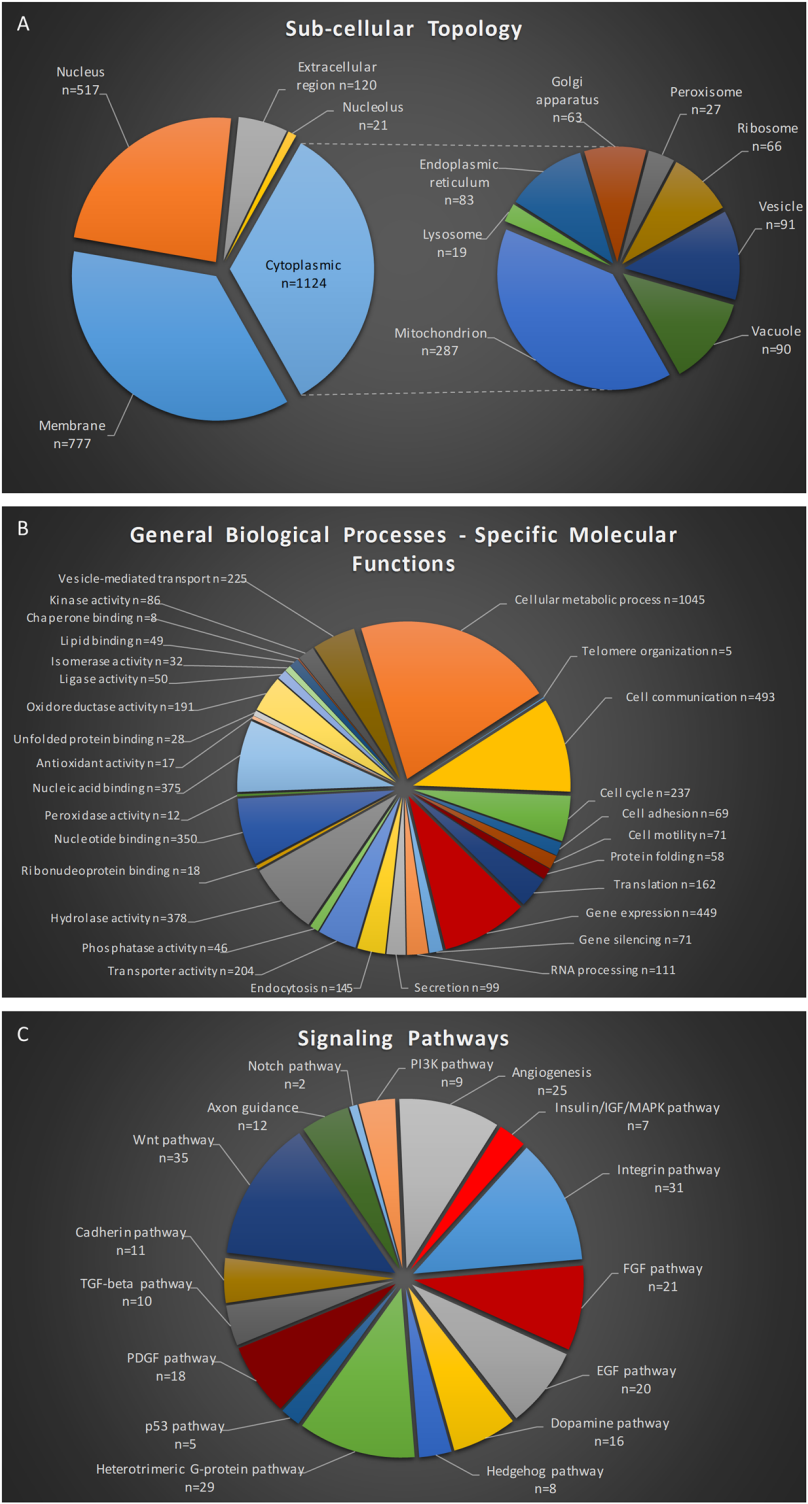
Gene Ontology-based annotation of single-transgenic elav.L-GAL4/+ *Drosophila* brain proteome. (**A**) Sub-cellular-topology classification of 2,335 fly-brain proteins having been obtained and identified via engagement of a high-resolution nLC-MS/MS proteomics technology. (**B**) Fly-brain proteome categorization in “Biological Processes” and “Molecular Functions”. (**C**) Major “Signaling Pathways” having been assembled in the fly-brain setting. Due to functional overlaps, a number of proteins are categorized in more than one group. (**A**,**B**) DAVID v.6.8 - Gene Ontology subroutine. (**C**) DAVID v.6.7 - PANTHER.

The elav.L-GAL4 is a strong neuronal cell-specific driver^51–53^, routinely used for gene-function analysis studies in *Drosophila* nervous system. Protein extracts from 100 manually dissected brains, derived from elav.L-GAL4 heterozygote *D. melanogaster* female flies (elav.L-GAL4/+) of 2 to 3 days old, were processed through a high-resolution nLC-MS/MS proteomics approach, and 7,663 unique peptides and 2,335 single proteins were identified (Supplementary Table 1). Out of this large collection of fly-brain proteins, 777 were classified to reside in the cell membranes (including external and internal ones), 538 in the nucleus (including nucleolus), 120 in the extracellular region and 1,124 in the cytoplasm (Fig. 1A). Furthermore, regarding cytoplasmic proteins, a number of organelle-related ones were recognized in the mitochondrion (n = 287), vesicle (n = 91), endoplasmic reticulum (n = 83), ribosome (n = 66), Golgi apparatus (n = 63), peroxisome (n = 27) and lysosome (n = 19) (Fig. 1A).

Next, we dissected the herein catalogued *D. melanogaster* brain-proteome contents into a plethora of general biological processes and specific molecular functions (Fig. 1B). The great majority of proteins seem to be implicated in metabolic processes (n = 1,045), cell communication (n = 493), gene expression (n = 449), hydrolase activity (n = 378), nucleic acid binding (n = 375), nucleotide binding (n = 350), cell cycle (n = 237), vesicle-mediated transport (n = 225) and transporter activity (n = 204) (Fig. 1B).

In an effort to reconstruct the fundamental pathways that critically control the physiology of neuronal cells, we analyzed *Drosophila* brain proteome via the PANTHER-pathway classification system being utilized through DAVID 6.7 bioinformatics resource (Fig. 1C). Proteins that belong to major signaling pathways and processes, such as axon guidance (n = 12) and angiogenesis (n = 25), which are crucial in neuronal-wiring mechanisms during brain development, were recognized. Pathways numerically enriched with brain proteins embrace the Wnt (n = 35), the integrin (n = 31), the FGF (n = 21), the EGF (n = 20) and the heterotrimeric G-protein pathway (n = 29). Interestingly, dopamine pathway, which besides the synthesis of neurotransmitter dopamine is also involved in functions such as learning, reward and motivation, is presented with 16 proteins (Fig. 1C).

The employment of liquid chromatography coupled with tandem-mass spectrometry has been previously employed to uncover the *Drosophila* head proteome of wild type flies^54^. In that study, a total number of 4,812 proteins were detected in an enriched membrane fraction of fly heads. More recently, Kuznetsova and collaborators analyzed the *Drosophila* brain proteome in a mixed population of equal male and female wild type flies of different ages, and identified 4,005 proteins^55^. Since the protein dataset of Kuznetsova and collaborators^55^ has been obtained from fly brains, albeit from diverse proteomic backgrounds, we, next, proceeded to brain proteome comparison, using UniProt accession and Flybase ID numbers, revealing approximately 1,727 shared proteins (Fig. S1). Without excluding technical differences of the employed protocols, the 608 unique proteins of our study may indicate an effect of GAL4 activity on the regulation of their expression.

**Figure 2:**
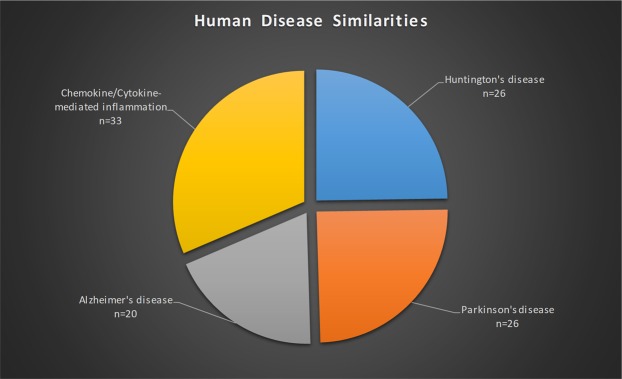
*Drosophila* brain-protein homologues to human counterparts can be critically implicated in neurological pathologies. The bioinformatics system employed was the PANTHER subroutine utilized through DAVID v.6.7.

Next, and since over 50% of fly genes show sequence homologies to human genes and approximately 75% of all known human-disease genes are believed to have functional fly homologues^1,3,7–9,56^, the identification in *Drosophila* brain proteome of proteins related to human pathologies was expected. Specifically, *Drosophila* brain protein-homologues of human ones associated with Parkinson’s disease (n = 26), Huntington’s disease (n = 26), Alzheimer’s disease (n = 20) and chemokine/cytokine-mediated inflammation (n = 33) were recognized (Fig. 2). These results highlight the prospect of utilizing *Drosophila* brain as a model system for the *in vivo* analysis of conserved pathways related to those being defective in human neurodegenerative diseases.

### UPS molecular reconstruction in *Drosophila* brain

**Figure 3:**
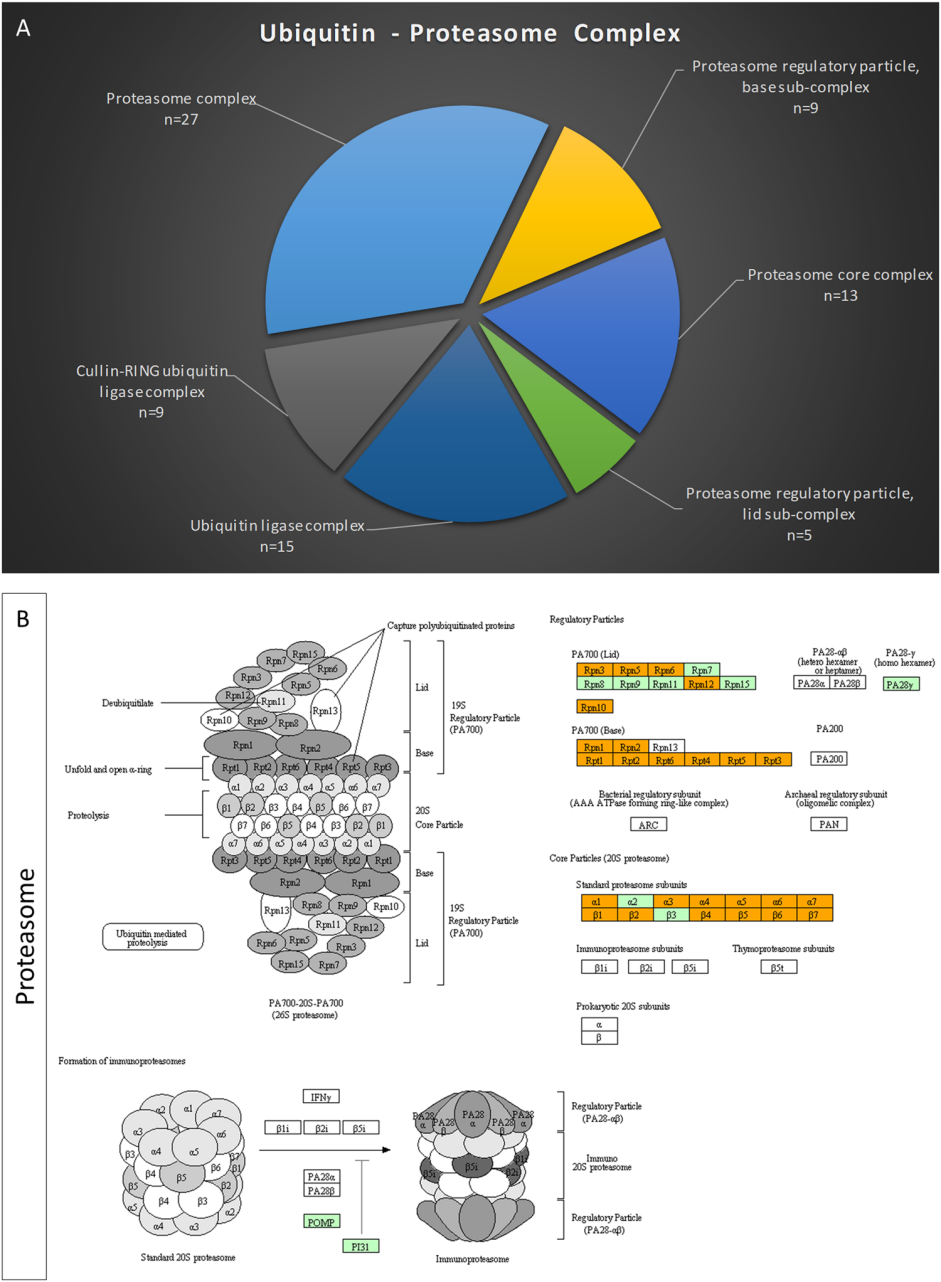
Reconstruction of UPS in *Drosophila* brain-proteome environment. (**A**) Categorization of fly-brain proteins strongly involved in UPS structure, function and regulation, following their characterization through engagement of the Gene Ontology subroutine of DAVID v.6.8. (**B**) Molecular reassembly of *Drosophila* 26S proteasome, through utilization of the KEGG-pathway database^46–48^ (permission ref: 200112). Orange-colored boxes: proteins of the present study identified in the fly-brain proteome. Green-colored boxes: proteins that were absent from our study (for either technical or biological reasons). White boxes: proteins that are completely missing from *Drosophila melanogaster* proteome.

Development of a number of neurodegenerative diseases is linked to failures in UPS, which result in the accumulation of neurotoxic proteins (reviewed in^1,3,4,22–24^). Deregulation of UPS affects the length and number of axons and dendrites, while it also leads to deficient synaptic function that is believed to be an early effect of neurodegenerative diseases, rendering UPS an important pharmacological target^4,20,57–62^. Thereby, we focused on proteins related to UPS components and regulators. A total of 27 proteins belonging to the proteasome complex were identified in our proteomic database, 13 of which were presented in the 20S core and the remaining 14 in the 19S regulatory complex (9 to base and 5 to lid sub-complex) (Fig. 3A). Additionally, 15 proteins being associated with the ubiquitin tagging of substrate-targets destined for proteasomal degradation were also recognized. Among them, 9 proteins were linked to cullin-RING E3 ubiquitin ligases (Fig. 3A), the largest superfamily of E3 ubiquitin ligases. Notably, molecular reconstruction of the 26S *Drosophila* brain proteasome through KEGG-pathway maps resulted in a rather complete protein model of both 20S and 19S proteasome complexes (Fig. 3B).

### Targeted disruption of 26S proteasome components in *Drosophila* neuronal tissues results in severe lifespan reduction and kinetic pathology

Based on the importance of UPS integrity to neuropathologies, we, next, investigated the effects of proteasome impairment in *Drosophila* neuronal cells during aging, with the use of GAL4/UAS genetic platform. GAL4/UAS, the most widely used system in *Drosophila* for achieving ectopic gene expression, allows the selective activation of any cloned gene or RNAi in a wide variety of tissue- and cell- specific patterns. Briefly, in one *Drosophila* line the GAL4 transcriptional activator of the yeast is introduced into its genome under the control of a cell/tissue-specific endogenous promoter, while in another line the target transgene or RNAi is cloned downstream of a UAS sequence. In the progeny of a cross between these lines, the target gene/RNAi of interest is expressed in the same cell/tissue-specific pattern as the GAL4 activator^63–65^. Hence, we genetically targeted critical 26S-proteasome subunits, using the neuronal-specific elav.L-GAL4 driver. Specifically, we examined representative subunits from both 19S-RP and 20S-CP, one of which (β5) carries proteolytic activity.

**Figure 4:**
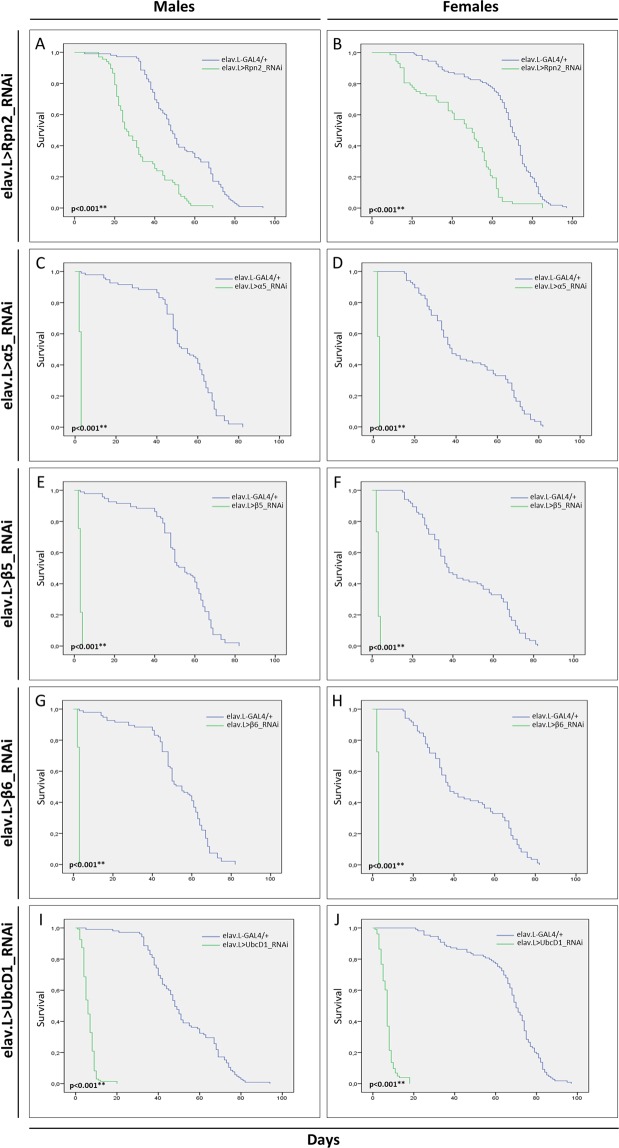
Neuronal cell-specific targeting of UPS key subunits in *Drosophila*. Lifespan profiles of *Drosophila* male (left panels) and female (right panels) transgenic fly populations, being characterized by RNAi-mediated targeting of the (**A**,**B**) Rpn2 19S-RP subunit (elav.L > Rpn2_RNAi), (**C**,**D**) α5 20S-CP subunit (elav.L > α5_RNAi), (**E**,**F**) β5 20S-CP catalytic subunit (elav.L > β5_RNAi), (**G**,**H**) β6 20S-CP subunit (elav.L > β6_RNAi) and (**I**,**J**) UbcD1 E2 enzyme (elav.L > UbcD1_RNAi), specifically in neuronal tissues. **p < 0.001.

**Figure 5:**
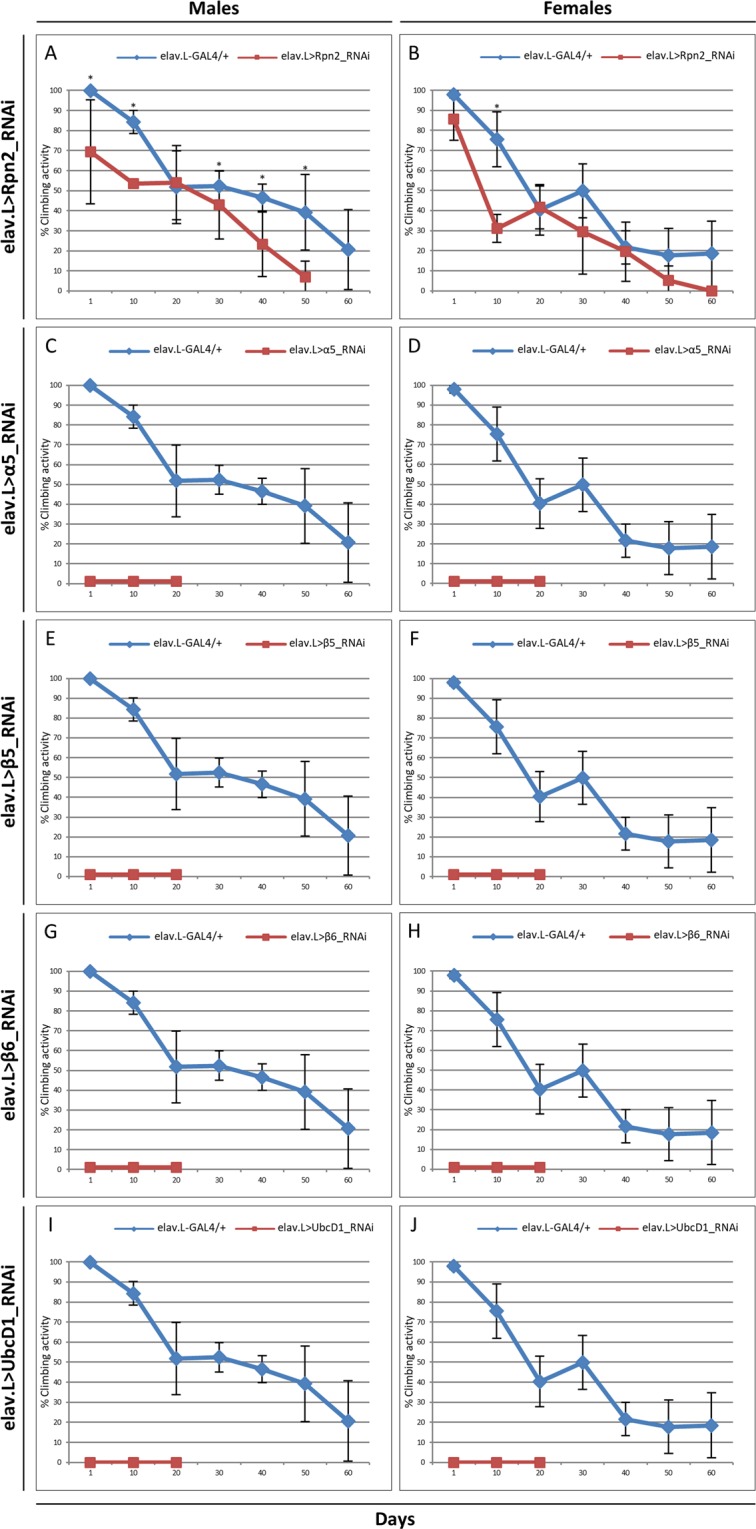
Genetic targeting of UPS in neuronal tissues causes climbing pathology in *Drosophila*. Climbing-activity patterns of male (left panels) and female (right panels) transgenic flies, carrying downregulated protein contents of the (**A**,**B**) Rpn2 19S-RP subunit (elav.L > Rpn2_RNAi), (**C**,**D**) α5 20S-CP subunit (elav.L > α5_RNAi), (**E**,**F**) β5 20S-CP catalytic subunit (elav.L > β5_RNAi), (**G**,**H**) β6 20S-CP subunit (elav.L > β6_RNAi) and (**I**,**J**) UbcD1 E2 enzyme (elav.L > UbcD1_RNAi), specifically in neuronal tissues. *p < 0.05.

According to previous reports, Rpn2 serves as scaffolding subunit of the 19S proteasome-base ring and together with Rpn1 comprise the two largest proteasome subunits of RP, which are both necessary for the coordinate functions of substrate shuttles, ubiquitin receptors and deubiquitinating enzymes^66–68^. After downregulating the Rpn2 subunit, specifically in neuronal cells, using the RNAi-based technology, a reduction of approximately 20 days in the viability of both male and female flies was observed at their 50% survival rate, as compared to control populations (Fig. 4A,B). Likewise, their climbing activity, especially in male flies, was also negatively affected, following an age-dependent declining pattern (Fig. 5A,B). Altogether, Rpn2 seems to represent an essential proteasome subunit controlling neuronal proteostasis. These findings are strongly supported by a previous report from our laboratory, describing the development of distinct, dysmorphic phenotypes in *Drosophila* eye and wing, in response to RNAi-mediated downregulation of Rpn1 or Rpn2 proteasome subunit^69^.

We, next, proceeded to investigate the role of outer α-rings of 20S-CP in normal nervous system. These are comprised of 7 different α subunits (α1–7), which provide the binding sites for 19S-RP and form the molecular gate responsible for regulating substrate entrance into the inner catalytic cavity of β-rings^28,70^. Flies with reduced levels of α5 proteasome subunit, specifically in neuronal cells, of both sexes, managed to survive only for few days (Fig. 4C,D), while they also presented dramatically diminished climbing activities (Fig. 5C,D), thus underscoring the indispensable contribution of α5 subunit to nervous-system physiological development and function in *Drosophila*. Similarly to Rpn2, the tissue-specific downregulated expression of α5 subunit in *Drosophila* has also been previously associated with developmentally malformed eyes and wings^69^. Interestingly, the critical role of α5 subunit in proteasomal activity has been demonstrated in yeast, with α5 mutations (such as the substitution of an α-pocket lysine residue) giving rise to proteasome holoenzymes that carry immature β subunits and reduced peptidase activities^71^. Strikingly, mutations in α5 subunit of *Saccharomyces cerevisiae* have proved to notably affect its lifespan and revealed that the 20S-CP gate opening is directly controlled by the α5 subunit^72^.

The inner β-rings are composed of the inactive (unable to hydrolyze) β3, β4, β6, and β7 subunits, and the active β1, β2 and β5 subunits, which contain proteolytic sites of different specificities. The β5 subunit with a chymotrypsin-like activity hydrolyzes proteins after hydrophobic amino acid residues and carries the major proteasome-related proteolytic activity^33,73^. We herein show that male and female flies being depleted of either β5 (Fig. 4E,F) or β6 (Fig. 4G,H) proteasome subunit, specifically in neuronal tissues, are presented with a life expectancy of only few days. Furthermore, they are characterized by total climbing (kinetic) deficiency, given their (both sexes) inability to pass the specified cylinder mark during the respective climbing assay (Fig. 5E–H). These observations are in line with previous ones, describing flies that bear a dominant-negative mutation in the *β6* gene, which die as undifferentiated pupae with failures in head eversion at a restrictive temperature^74^. This mutation has been also reported to alter cell-fate determination in the adult *Drosophila* sense-organ lineage^75^. It has been previously proposed that a deficient β6-proteasome subunit may disrupt proteasomal activity by altering the β2-β6 interfacing regions^75,76^. Importantly, previous studies have associated β5 and β6 proteasome-subunit deregulation with disrupted *Drosophila* eye and wing development^69,77,78^. In accordance to our findings, data from transgenic mice have shown that reduction in β5-associated chymotrypsin-like activity leads to multiple early-aging phenotypes and shortened lifespan^79^. On the contrary, overexpression of β5 subunit can improve proteostasis during aging and can increase longevity in *Drosophila*^80^. Ιt seems that certain thresholds of 26S-proteasome activities are required for *Drosophila* nervous-system physiological development and function.

### RNAi-mediated downregulation of the E2 ubiquitin-conjugating enzyme UbCD1 specifically in *Drosophila* neuronal tissues compromises longevity and climbing activity

Ubiquitination of substrates destined for proteasomal degradation depends on the sequential actions of ubiquitin-activating enzymes (E1s), ubiquitin-conjugating enzymes (E2s) and ubiquitin-ligases (E3s), which mediate the final transfer of ubiquitin to the selected substrates^81^. E2s contain a highly conserved 150–200 amino acid ubiquitin-conjugating catalytic-core domain and are involved in the transfer of ubiquitin or ubiquitin-like proteins to downstream conjugation targets. E2s are responsible for the selection of correct modifier, the determination of length, type and topology of attached ubiquitin chain, and the processivity of chain-assembly reaction, thereby defining the fate assignment of the modified protein^73,81–84^. It has recently emerged that E2s, besides their typical role in protein degradation, are also involved in processes controlling protein function, sorting and localization, while their aberrant regulation has been associated with many disease pathways^81,85^. Among them, *UbcD1*, or *effete*, encodes a highly conserved E2 enzyme, with approximately 80% sequence identity to the yeast *Ubc4*/*Ubc5* homologue, which mediates the selective protein-ubiquitination and degradation processes^86^. UbcD1 has been also associated with proper telomere behavior during cell division^87^, Hedgehog signaling by controlling Ci stability^88^, female germline stem-cell maintenance^89^, and regulation of apoptosis through its involvement in the poly-ubiquitination and degradation of DIAP1^90,91^. UbcD1 has been directly associated with *Drosophila* SCF complex of proteasome-dependent proteolysis^92–94^ and, as it is illustrated in Fig. 3A, the cullin-RING E3 ligase (SCF) complex can be readily detected in *Drosophila* nervous system.

Therefore, in an *in vivo* attempt to prove the importance of neuronal-specific UbcD1 targeting to the lifespan and climbing activity of *D. melanogaster* during aging, we genetically downregulated its expression using the elav.L-GAL4 and UbcD1_RNAi transgenic flies. *UbcD1*-targeted male (Fig. 4I) and female (Fig. 4J) flies were presented with a maximum life expectancy of only 20 days, while their vast majority were dying within the first 10 days. Notably, both male (Fig. 5I) and female (Fig. 5J) fly populations appeared with completely impaired climbing activities, since none of the tested flies proved capable to climb over the specified cylinder mark. Similarly, another study has reported that among 16 genes encoding E2 enzymes, only the RNAi-mediated *UbcD1* targeting resulted in neuroblast overgrowth^94^.

### Protein deubiquitination deregulation in neuronal cells during *Drosophila* aging

Ubiquitination, the post-translational modification of proteins by ubiquitin, can be reversed by deubiquitinating enzymes (DUBs), a specific family of proteases that catalyze the removal of ubiquitin (or ubiquitin-like) molecules from target substrates, modulating protein stability and signaling^31,33^. DUBs are divided into 6 structurally distinct subfamilies based on the architecture of their catalytic domains and mechanisms of action^31,33,95,96^. In the human genome, approximately 100 DUBs are encoded, while in the fly there have been identified around 45-family members^31,95,97,98^. DUBs serve as key regulators of several intracellular processes, such as NFκB activation (USP11 and 15), c-Myc stability (USP28), p53 stability (USP2a, 7 and 10), DNA-damage response (USP1) and apoptosis (USP7 and 28)^27,31,95,96,99^. Aberrant expression patterns and mutant forms of DUBs have been implicated in pathogenesis of several human diseases, including cancer and neurological pathologies^31,95,96,98^, and, as such, DUBs have emerged to represent attractive drug targets for pharmacological intervention strategies^27,29,99^.

Ubiquitous suppression of most DUBs in *Drosophila* has been associated with severe deregulation of fly development, motility and longevity^98^. Hence, in an effort to *in vivo* examine the significance of deubiquitination process in neuronal tissues during *Drosophila* aging, we herein generated transgenic flies carrying downregulated expression of the dUBP64 protease (a deubiquitinating enzyme). Usp47, which is the human ortholog of the fly dUBP64 deubiquitinating enzyme, has been found to interact with the E3 ubiquitin ligase SCF^100^ and to play a key role in the control of axonal growth during neuronal development^101^, while its depletion from different cell lines has been associated with decreased cell survival^100^. In *Drosophila*, dUBP64 functions as a modifier of position-effect variegation^102^ and controller of cell-fate decision during eye development, by regulating the transcriptional repressor tramtrack^103^.

**Figure 6:**
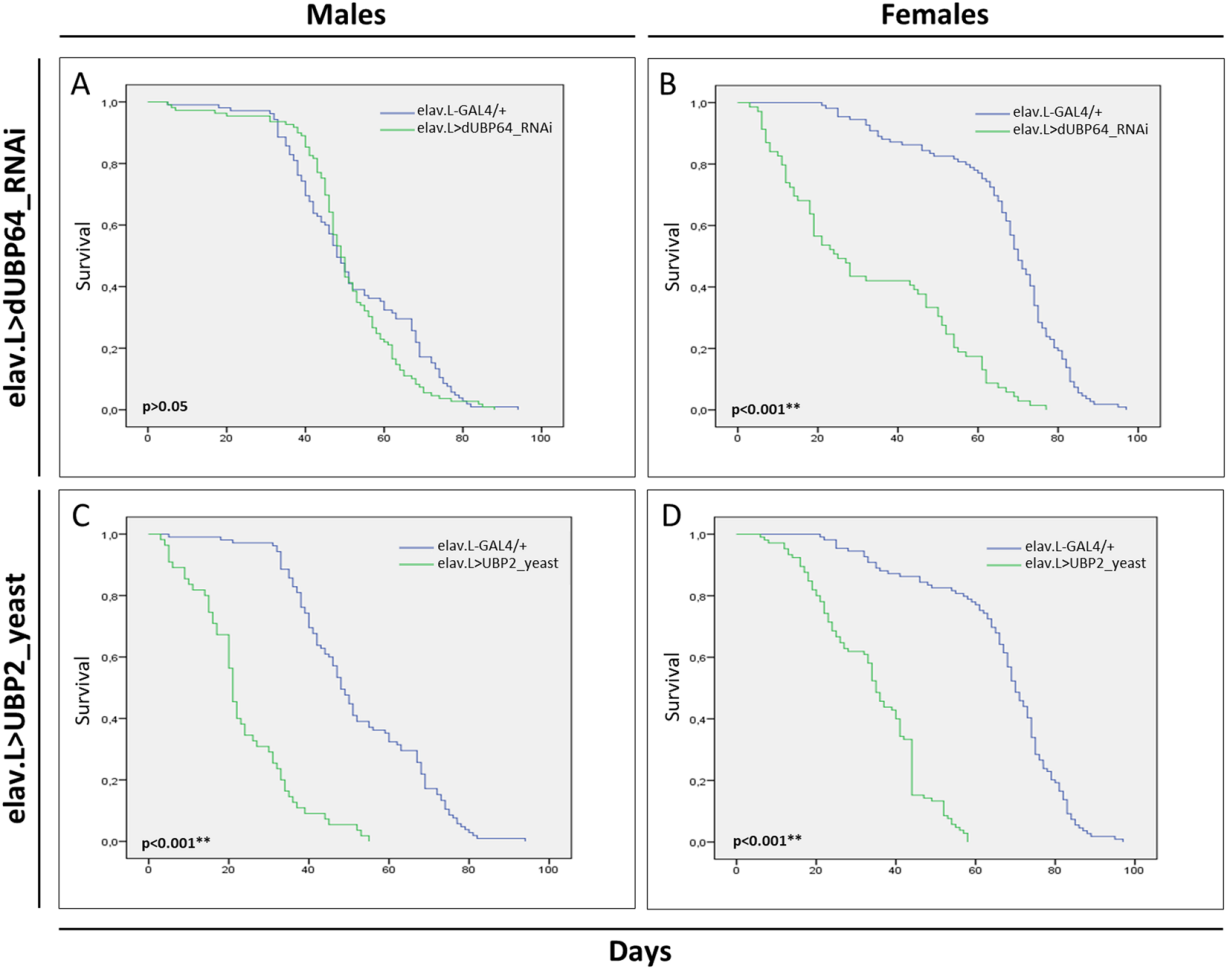
Genetic deregulation of ubiquitinated protein-load homeostasis in *Drosophila* neuronal tissues. Fly-longevity profiles of male (left panels) and female (right panels) transgenic flies, bearing either downregulated protein contents (via employment of RNAi technology) of the (**A**,**B**) dUBP64 deubiquitinating enzyme (elav.L > dUBP64_RNAi), or elevated (overexpressed) protein levels of the (**C**,**D**) yeast UBP2 deubiquitinating enzyme (elav.L > UBP2_yeast), specifically in neuronal tissues. **p < 0.001.

**Figure 7:**
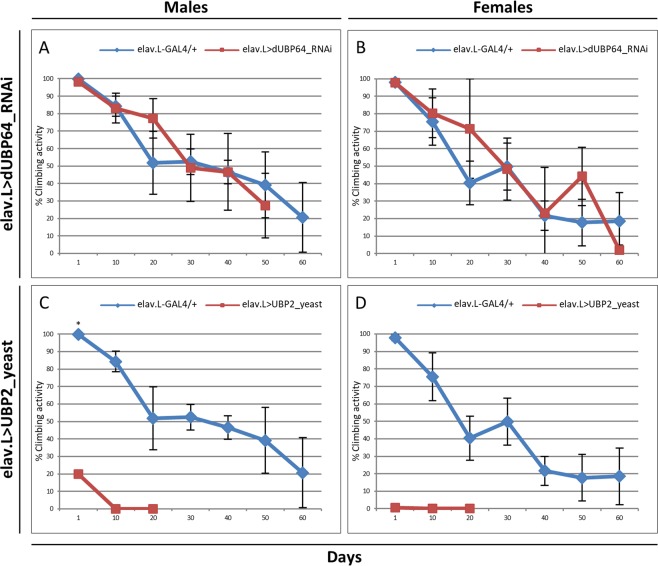
Effects of neuronal-specific deregulation of protein deubiquitination process in *Drosophila* climbing. Climbing-activity patterns of male (left panels) and female (right panels) transgenic flies, carrying either downregulated protein contents of the (**A**,**B**) dUBP64 deubiquitinating enzyme (elav.L > dUBP64_RNAi), or increased protein levels of the (C,D) yeast UBP2 deubiquitinating enzyme (elav.L > UBP2_yeast), specifically in neuronal tissues. *p < 0.05.

In contrast to male transgenic flies (Fig. 6A), female populations, at their 50% survival rate, were presented with a reduction of approximately 40 days in their viability, as compared to control populations (Fig. 6B). Moreover, both male and female *dUBP64*-targeted flies were characterized by similar to controls climbing activities (Fig. 7A,B). Similar fly-sex-specific differential longevity profiles have been also reported in response to pathogen infection and altered mitochondrial dynamics^104,105^. Furthermore, to investigate the effects of DUB overexpression in lifespan and climbing activity, transgenic flies overexpressing the -yeast- UBP2 protease (a deubiquitinating enzyme), specifically in neuronal tissues, were generated through utilization of the elav.L-GAL4 driver. Both male (Fig. 6C) and female (Fig. 6D) transgenic flies were presented with significantly reduced viabilities and climbing activities (Fig. 7C,D), as compared to control populations. It seems that UBP2 overexpression critically compromises *Drosophila* longevity and climbing activity, and that neuronal cells are unable to counterbalance, or neutralize, the proteotoxic stress being induced by UBP2 abundance. Ubiquitination pathway is intrinsically required for the regulation of synaptic growth and function in *Drosophila*, with the -yeast- UBP2 deubiquitinating enzyme overexpression specifically in nervous system leading to synaptic overgrowth and defect in neurotransmitter release^106^. Overexpression of UBP2 has been also associated with synaptic-elimination delay in postsynaptic muscles^107^. Axon and dendrite pruning are fundamental for development of proper circuitry in *Drosophila* nervous system, and UBP2 elevated contents can cause blockage of both axon and dendrite pruning, and degeneration^108–110^.

Overall, we herein mapped, in a high-resolution scale, the brain proteomic landscape of the single-transgenic elav.L-GAL4/+ *D. melanogaster* fly strain. This, together with the availability of fly-mutant (genetic) lines able to model some aspects of human neuropathology (reviewed in^1,7,56,111^), may enable the screen of a large number of drugs, which are expected to be favorably exploited for further elucidating disease mechanisms, and identifying novel druggable targets and disease therapies. Indeed, researchers have recently used genetically modified *Drosophila* flies, as a personalized biological platform, for discovering the best (optimum) drug therapy being applied to a patient carrying treatment-resistant colorectal cancer^112^. Conclusively, fine tuning of ubiquitinated-protein homeostasis in neuronal cells has herein proved essential for animal’s well-being during aging.

## Supplementary information

Supplementary Figure S1 and Table S1 caption.

Supplementary Table S1.
