# Supplementary material for: Proteomic mapping of Drosophila transgenic elav.L-GAL4/+ brain as a tool to illuminate neuropathology mechanisms
Source: Sci Rep. 2020 Mar 25;10:5430. doi: 10.1038/s41598-020-62510-0 (PMC7096425; doi:10.1038/s41598-020-62510-0)
Supplement: Supplementary file 1 — Supplementary Figure S1 and Table S1 caption. [file 41598_2020_62510_MOESM1_ESM.pdf]

Supplementary information

Proteomic mapping of *Drosophila* transgenic elav.L-GAL4/+ brain as a tool to illuminate neuropathology mechanisms

Athanassios D. Velentzas[1], Stamatia A. Katarachia[1], Niki E. Sagioglou[1], Maria M. Tsioka[1], Athanasios K. Anagnostopoulos[2], Vassiliki E. Mpakou[1], Eleni I. Theotoki[1], Aikaterini F. Giannopoulou[1], Konstantinos E. Keramaris[3], Issidora S. Papassideri[1], George Th. Tsangaris[2] and Dimitrios J. Stravopodis[1,*]

[1]Section of Cell Biology and Biophysics, Department of Biology, School of Science, National and Kapodistrian University of Athens (NKUA), Athens, Greece

[2]Systems Biology Center, Biomedical Research Foundation of the Academy of Athens (BRFAA), Athens, Greece

[3]Department of Hematology and Bone Marrow Transplantation, Medical School, National and Kapodistrian University of Athens (NKUA), Athens, Greece

[*]**Corresponding Author**:

Dimitrios J. Stravopodis, *BSc*, *MSc*, *PhD*, Associate Professor, Section of Cell Biology and Biophysics, Department of Biology, School of Science, National and Kapodistrian University of Athens (NKUA), Panepistimiopolis, Zografou 157 01, Athens, Greece. Tel.: +30 210 727 4105, Fax: +30 210 727 4742, E-mail: dstravop@biol.uoa.gr

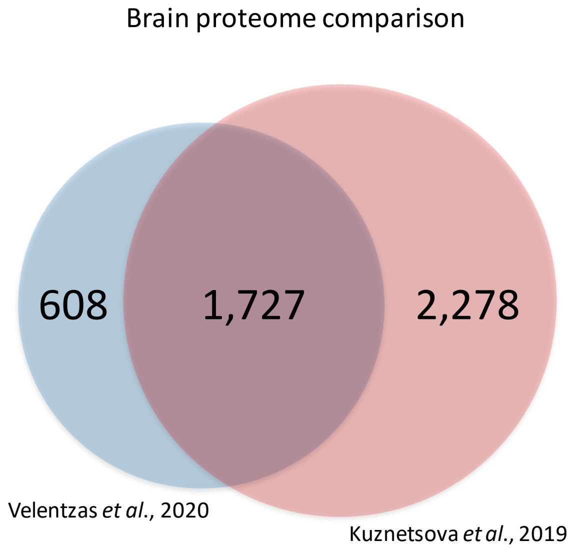

Figure S1

***Drosophila* brain proteome overlap between elav.L-GAL4/+ transgenic flies and wild type ones[1]**. Venn's diagram depicting the numbers of unique and overlapping brain protein profiles among elav.L-GAL4/+ transgenic flies (current study) and wild type ones (Canton S line)[1], after the comparison of their respective UniProt accession numbers. Since several UniProt accession numbers of *Drosophila* proteins are redundant, we also converted the unique accession numbers to Flybase IDs, with the use of UniProt's "Retrieve/ID mapping" tool, and compared again. The illustrated diagram is the result of both comparisons. Nevertheless, some outcomes may be underestimated.

Supplementary Table S1 caption

High-resolution mapping of *Drosophila* brain-proteomic landscape. Protein library (n=2,335 single members) derived from single-transgenic elav.L-GAL4/+ *Drosophila melanogaster* adult (young female; 2-3-day old) brains, specifying, for each obtained and identified fly-brain protein (via nLC-MS/MS), the following parameters: (A) UniProt accession number, (B) name and description, (C) Mascot score and (D) sequence coverage. Mascot score serves as a reliable parameter for protein quantification analysis, as previously reported[2]. Additional important features of the nLC-MS/MS proteomics analysis, such as the number of unique peptides, the number of amino acid residues (AAs), the molecular weight (MW) (kDa) and the isoelectric point (pI), are also described.

References

1 Kuznetsova, K. G. *et al.* Brain Proteome of Drosophila melanogaster Is Enriched with Nuclear Proteins. *Biochemistry (Mosc.)* **84**, 71-78, https://doi.org/10.1134/S0006297919010097 (2019).

2 Mallick, P. & Kuster, B. Proteomics: a pragmatic perspective. *Nat Biotechnol* **28**, 695-709, https://doi.org/10.1038/nbt.1658 (2010).
